# Dietary 3'-sialyllactose reduces sensitization and type 2 inflammation in a house dust mite induced acute allergic asthma model

**DOI:** 10.3389/falgy.2025.1604917

**Published:** 2025-12-01

**Authors:** M. Zuurveld, J. W. M. de Kleer, I. van Ark, A. Leusink-Muis, A. I. Kostadinova, J. Garssen, G. Folkerts, B. van’t Land, L. E. M. Willemsen

**Affiliations:** 1 Division of Pharmacology, Utrecht Institute for Pharmaceutical Sciences, Faculty of Science, Utrecht University, Utrecht, Netherlands; 2 Danone Research and Innovation, Utrecht, Netherlands; 3 Center for Translational Immunology, University Medical Center Utrecht, Utrecht, Netherlands

**Keywords:** allergic asthma, human milk oligosaccharides, immunomodulation, sialylated oligosaccharides, allergy prevention

## Abstract

**Introduction:**

Allergic asthma affects over 300 million people globally, characterized by a type 2 immune response to allergens like house dust mite (HDM). This includes eosinophilia, IgE production, and symptoms such as bronchial hyperresponsiveness. Human milk oligosaccharides (HMOS), ingested by breastfed infants, have immunomodulatory effects and may help prevent allergic diseases, like asthma.

**Methods:**

This study investigates the effects of two sialylated HMOS, 3′-sialyllactose (3′SL) and 6′-sialyllactose (6′SL), in a murine model of HDM-induced allergic asthma. Male BALB/c mice (6–7 weeks old) were fed an AIN93G diet with or without 0.1% or 0.5% 3′SL or 6′SL from 2 weeks before HDM sensitization until sacrifice. Airway hyperresponsiveness was measured after the final HDM challenge, and broncho-alveolar lavage fluid (BALF) and lung tissue were collected for analysis.

**Results:**

Dietary 0.5% 3′SL, 0.1% 6′SL, or 0.5% 6′SL prevented methacholine-induced airway hyperresponsiveness in HDM-challenged mice compared to control diet. Mice fed the 0.5% 3′SL diet had elevated SCFA levels in cecum content. Both 3′SL and 6′SL groups showed reduced HDM-induced macrophage influx in BALF. Mice on 3′SL diets had lower total inflammatory cell influx, while those on 0.5% 6′SL had increased eosinophils in BALF, associated with higher IL33, TNFα, CCL5, IFNγ levels, and reduced regulatory T cells. The 3′SL diets also prevented increases in HDM-specific IgE and mMCP1 in serum.

**Conclusion:**

Dietary 3′SL and 6′SL showed dose-dependent, differential clinical and immunological outcomes in HDM-sensitized mice. Both 0.5% 3′SL, 0.1% 6′SL, and 0.5% 6′SL reduced airway hyperresponsiveness. However, 0.5% 6′SL increased eosinophilic inflammation, while 3′SL protected against HDM-induced sensitization and asthma development.

## 1 Introduction

Allergic asthma is increasingly common in Western societies, with over 300 million people affected worldwide (1). The underlying allergic response is characterized by a type 2 immune response to airborne allergens, such as pollen, animal dander, fungi and house dust mite (HDM) (2). Uptake of allergens by antigen-presenting cells (APCs) occurs through sampling of the airway lumen by dendritic cells (DCs) and/or through crossing of the allergen into the lamina propria. Following uptake of the allergen, DCs travel to secondary lymphoid tissue to present the allergen to naive Th cells which leads to differentiation into Th2 cells (3). These Th2 cells activate B cells, which leads to class switching towards immunoglobulin E (IgE) producing plasma cells. This excreted allergen-specific IgE binds to the high affinity receptor (FcεRI) present on mast cells (MCs). Upon a subsequent encounter with the allergen, cell bound allergen-specific IgE crosslinks, inducing mast cell degranulation (4, 5). Degranulation involves the release of proinflammatory mediators present in mast cell granules, inducing vasodilation, bronchoconstriction, and increased capillary permeability, resulting in clinical symptoms (6).

The WHO recommends exclusive breastfeeding for the first 6 months of life, as it has various benefits for both mother and child (7). Breastmilk consists of the necessary nutrients and bioactive compounds to provide appropriate nutrition for the infant (8, 9). It is a dynamic fluid, which composition changes depending e.g., on the stage of lactation, the mother's ethnicity, diet, age, and even the time of the day and the period since the last feed (10, 11). Breastfed children ingest high concentrations of human milk oligosaccharides (HMOS), as HMOS are the third largest solid component of breastmilk. All mammalian milk contains oligosaccharides, however human milk contains a uniquely high concentration and complex structures (12–14). Some of these have a prebiotic function, meaning that they can influence the microbiome which in turn can influence the resilience and maturation of the immune system. In addition to the microbiome dependent effects, some of the oligosaccharides can influence immune cells directly, as was observed e.g., by the immunomodulatory and tolerogenic effects observed after dendritic cells were *in vitro* exposed to isolated total HMOS (15). The immunomodulatory effects induced by HMOS may therefore be involved in the prevention of the onset of allergic diseases such as allergic asthma (5, 16).

Sialylated oligosaccharides are abundantly present in mammalian milk (17). Sialylated HMOS, such as 3′-sialyllactose (3′SL) and 6′-sialyllactose (6′SL), are thought to affect several biological functions, including supporting the neonatal developing immune system (18). Studies have shown that both 3′SL and 6′SL promote *in vitro* differentiation of intestinal epithelial cells (19–22) and decrease the incidence and severity of diarrhea in piglets (23). These effects are partially explained by binding of 6′SL to G protein-coupled receptor 35 (GPR35) (24) as well as promoting growth of beneficial bacteria such as *Bifidobacteria* and *Bacteroides* strains by both 3′SL and 6′SL (25, 26), however interactions with several immune related receptors (e.g., TLR4 and Siglecs) have been described (16). The latter is reflected by increased production of short chain fatty acids (SCFAs), as utilization of 3′SL and 6′SL occurs in high levels by these bacterial species (27). Increased levels of specific SCFAs are linked to improved allergic outcomes both in *in vivo* preclinical models as well as in children (28, 29). In addition to the local effects in the intestines, sialylated HMOS are suggested to be systemic immunomodulators as well. For instance, 3′SL was found to decrease leukocyte adhesion to endothelial cells (30), while binding of 3′SL to TLR4 on dendritic cells strengthened both Th1 and Th17 immunity (31).

Previously, it was demonstrated that dietary prebiotics, such as galacto- and fructo-oligosaccharides (GOS and FOS), and their fermentation products, like SCFAs, can positively influence the immunological outcomes in HDM-induced allergic asthma in murine models (28, 32). Recently manufactured HMOS have become available and amongst others 6′SL was observed to improve allergic symptoms in murine models of food allergy or allergic asthma (33, 34). In the current study the immunomodulatory effects of two common and structurally similar sialylated HMOS, 3′-sialyllactose (3′SL) and 6′-sialyllactose (6′SL), were compared in a murine model for acute house dust mite induced allergic asthma.

## 2 Materials and methods

### 2.1 Diet preparation

The HMOS 3′-sialyllactose (3′SL) and 6′-sialyllactose (6′SL) were purchased from Jennewein Biotechnlogie GmbH (Germany). Experimental diets were based on an AING93-G diet, supplemented (Sniff Spezialdiëten GMBH, Germany) with 0.1% or 0.5% 3′SL or 6′SL, and isocalorically compensated with cellulose. Animals had *ad libitum* access to food and water.

### 2.2 Animals

Six to seven-week-old male BALB/cAnNCrl mice (Charles River) were housed in individually ventilated cages under a 12 h/12 h light/dark cycle, controlled relative humidity (50%–55%) and temperature (21 ± 2°C) conditions. Mice were randomly divided into experimental groups (*n* = 6 for Sham, *n* = 12 for HDM sensitized and challenged groups) and received intervention diets upon arrival. Cage enrichment consisted of woodchipped bedding, wood-curls as nesting material and a plastic shelter. This study was conducted in accordance with institutional guidelines for the care and use of laboratory animals of the Utrecht University, and all animal procedures were approved by the local Animal Welfare Body under an Ethical license provided by the national competent authority (Centrale Commissie Dierproeven, CCD), securing full compliance the European Directive 2010/63/EU for the use of animals for scientific purposes.

### 2.3 Animal procedures and airway hyperresponsiveness

**Figure 1:**
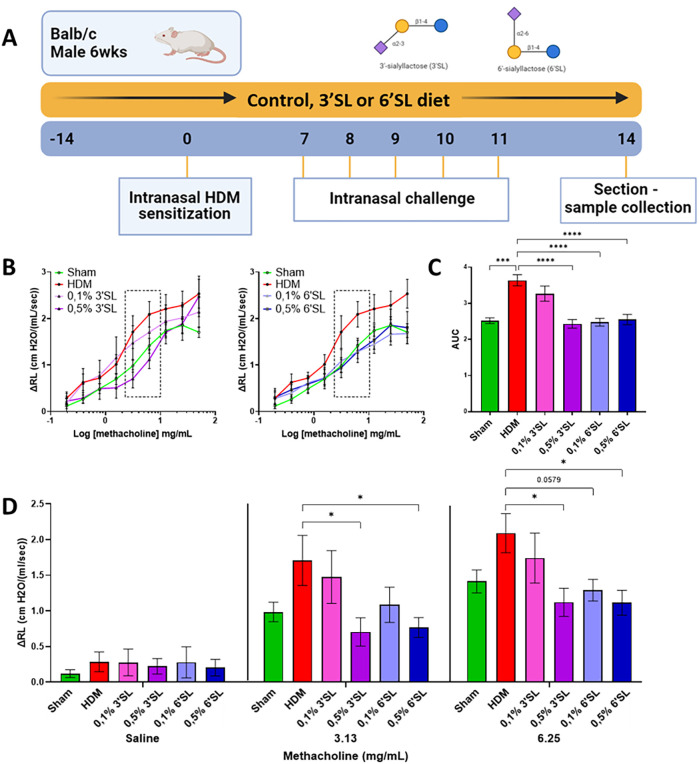
**(A)** A schematic overview of the experimental design, including dietary intervention with 3′SL and 6′SL supplemented diets fed from 14 days prior to and during intranasal sensitization and challenge with HDM. 72 h after the final challenge, mice were sacrificed and samples for analysis were collected. **(B)** The increase in airway resistance in response to progressive doses of methacholine was measured in ventilated anesthesized mice. The values inside the dotted boxes are separately displayed in **(D)**. **(C)** The area under the curve was calculated of the increased airway resistance was calculated and **(D)** the airway resistance in response to saline, 3.13 mg/mL and 6.25 mg/mL methacholine are shown. Data is presented as mean ± SEM of 6–12 animals per group. Sham and HDM-allergic groups were statistically compared using an unpaired *t*-test. Dietary intervention groups were compared to the HDM-allergic group by One-Way ANOVA followed by a Dunnett's *post hoc* test (*p < 0.05, **p < 0.01, ***p < 0.001, ****p < 0.0001).

An overview of the experimental design is given in Figure 1A. 14 days after arrival, mice were intranasally sensitized with 1 μg HDM (Greer Laboratories, USA) in 40 μL PBS under isoflurane anaesthesia. From days 21 to 25, mice were daily challenged intranasally with 10 μg HDM in 40 μL PBS. 72 h after the final challenge dynamic airway hyperresponsiveness to increasing doses of methacholine was measured under terminal anaesthesia (Flexivent, Scireq, France), subsequently mice were sacrificed by intraperitoneal overdose of pentobarbital (600 mg/kg, Nembutal™, Ceva Santé Animale, The Netherlands) and samples were collected for further analysis.

### 2.4 Bronchoalveolar lavage (BAL)

After sacrifice, lungs were lavaged with 1 mL pyrogen-free saline (0.9% NaCl, 37°C) supplemented with protease inhibitors (Complete Mini, Roche Diagnostics, Germany). The lungs were lavaged three more times with 1 mL saline solution (0.9% NaCl, 37°C). BALF cells were centrifuged (400 × g, 5 min) and the pellets were collected. Total cell count was determined using a Bürker-Türk chamber (magnification 100×). Cytospin preparations were stained with Diff-Quick (Merz & Dade A.G., Switzerland) for differential BAL cell count.

### 2.5 Serum analysis

Blood was collected after sacrifice via eyeball extraction in a Minicollect serum tube (Greiner Bio-One B.V., Netherlands). Collected blood was kept at room temperature and centrifuged for 10 min at 18,000 g. Serum was collected and stored at −20°C until further analysis.

### 2.6 Preparation of lung homogenates

Lungs were homogenized in 1% Triton ×100 (Sigma-Aldrich) in PBS containing protease inhibitors (Complete Mini, Roche Diagnostics) using a Precellys Tissue Homogenizer and Precellys homogenizer tubes (Bertin, USA). Homogenates were centrifuged at 18,000 g for 10 min. Supernatant was collected and stored at −20 °C for cytokine analysis.

### 2.7 Flow cytometric analysis of lung tissue

Lung tissue was collected after sacrifice and enzymatic digested using a buffer containing DNase I and Collagenase A (Roche Diagnostics, Switzerland). Fetal calf serum (FCS) was added to stop the digestion after 30 min. The lung tissue was passed through a 70 μm filter to obtain single cell suspensions. Cell suspension were incubated for 4 min on ice in red blood cell lysis buffer (4.14 g NH_4_Cl, 0.5 g KHCO_3_, 18.6 mg NA_2_EDTA in 500 mL demi water, sterile filtered, pH 7.4) Lysis was stopped by adding FCS. Lung cells were washed with RPMI 1640 (Lonza, USA).

5 × 10^5^ cells were used for extracellular staining, 1 × 10^6^ cells were used for intracellular staining. Cells were first stained with Fixable Viability Dye eFluor780 for 30 min. Nonspecific binding was blocked using antiCD16/CD32 blocking buffer. Subsequently samples were stained for 30 min at 4°C using titrated amounts of the following antibodies: CD4-BV510, CD69-PE-Cy7, CXCR3-PE, T1ST2-FITC, CD25-PerCP Cy5.5, CD127-PE Vio770, FoxP3-FITC, RORγt-AF647, CCR6-PE, LAP-BV421. To allow intranuclear staining of transcription factors, cells were permeabilized using FoxP3/Transcription Factor staining buffer set (eBioscience, USA) following the manufacturer's instructions. FACS Canto II (BD Biosciences) was used to measure stained samples and obtained data was analysed using Flowlogic Software (Inivai Technologies, Australia). The gating strategy using a representative sample is given in Supplementary Figure S2.

### 2.8 ELISA

To measure serum HDM-specific IgE, IgG1 and IgG2a levels, high binding 96 wells plates (Corning Costar) were coated with HDM (10 µg/mL) and incubated overnight at 4°C. After blocking with 1% BSA in PBS, plates were washed and serum samples were added to incubate for 2 h. Serum samples had to be diluted prior to incubation, all samples were equally diluted and measured on the same plate. Plates were washed again and 1 µg/mL biotin anti-mouse IgE, IgG1 or IgG2a (BD Biosciences) was added for 1.5 h. Plates were incubated with streptavidin-HRP for 30 min, followed by addition of a substrate solution. Washing steps were performed in between. Reaction was stopped by addition of 2M H2SO4, and absorption was measured at 450 nm.

Serum mMCP1 concentrations, and lung homogenate supernatant concentrations of CCL5, CCL20, CCL22 (R&D systems, USA) IL5, IL13, IL33, IFNγ, IL17, IL10, TGFβ and TNFα (Invitrogen, USA) were measured according to the manufacturer's instructions. Cytokine concentrations in lung homogenates were calculated per mg protein in the homogenate supernatant.

### 2.9 Short chain fatty acid levels in cecum content

Cecum contents were collected and stored in −80°C until further use. After thawing, samples were weighed and 5× diluted with ice cold PBS. 1.0 mm glass beats (BioSpec, USA) were added and samples were vortexed for 90 s to allow homogenization. Homogenized samples were centrifuged for 10 min at 18,000 g at 4°C, supernatants were collected and stored at −80°C until further analysis. SCFAs were detected by gas chromatography as previously described (35).

### 2.10 Statistical analysis

Statistical analyses were performed using Graphpad Prism (Version 9.4.1) software. Data was analyzed using an unpaired *t*-test to compare the Sham and OVA groups. All intervention groups were compared to the OVA group using One-way ANOVA followed by Dunnett's multiple comparisons *post hoc* test. If data did not fit a normal distribution, a logarithmic transformation was applied prior to further analysis. *p* < 0.05 was considered statistically significant, data is represented as mean ± SEM. The Sham group contained *n* = 6 and the HDM sensitized and challenged groups had *n* = 12 mice per group.

## 3 Results

### 3.1 Dietary 3′SL and 6′SL prevent the development of airway hyperresponsiveness

To study the effects of dietary 3′SL or 6′SL in HDM sensitized and challenged mice, airway hyperresponsiveness (increased airway resistance) in response to inclining concentrations of methacholine (displayed as log concentration) was assessed (Figure 1B). Supplementary Figure S1 shows increased airway hyperresponsiveness in response to methacholine on a linear axis. The area under the curve (AUC) was calculated (Figure 1C). The AUC of increased airway resistance as measured in response to methacholine was moderately but significantly higher in HDM sensitized and challenged mice compared to PBS sensitized and saline challenged mice (Sham). AUC of airway hyperresponsiveness in mice fed 0.5% 3′SL, 0.1% 6′SL or 0.5% 6′SL diets was significantly lower compared to the HDM sensitized and challenged mice fed a control diet (Figure 1C). At baseline (Saline, Figure 1D) no significant differences were observed between the groups. Airway resistance was significantly lower in HDM sensitized and challenged mice fed the 0.5% 3′SL or 0.1% 6′SL or 0.5% 6′SL diets compared to HDM sensitized and challenged mice fed control diet when exposed to 3.13 and 6.25 mg/mL methacholine (Figure 1D). These data show that dietary intervention of 3′SL and 6′SL prevent the development of airway hyperresponsiveness.

### 3.2 Dietary 0.5% 3′SL increases SCFA levels in cecum content

**Figure 2:**
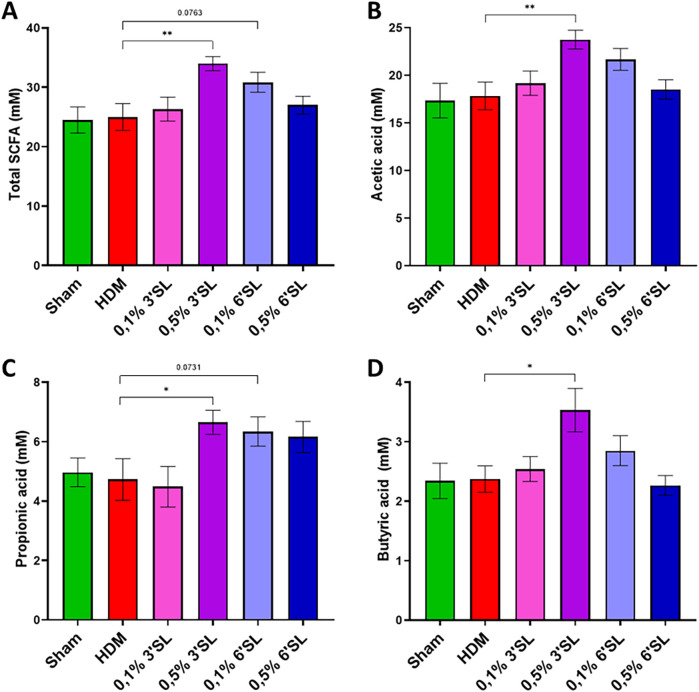
SCFA concentrations were determined in cecum content. **(A)** Total SCFA, **(B)** acetate, **(C)** propionate and **(D)** butyrate concentrations are displayed. Data is presented as mean ± SEM of 6-12 animals per group. Sham and HDM-allergic groups were statistically compared using an unpaired *t*-test. Dietary intervention groups were compared to the HDM-allergic group by One-Way ANOVA followed by a Dunnett's *post hoc* test (*p < 0.05, **p < 0.01).

SCFAs are produced upon utilization and fermentation of prebiotic fibres in the cecum of the mice and were therefore measured in cecum content. Total SCFA levels (acetate, propionate, butyrate, iso-butyrate, valerate and iso-valerate) in cecum content were elevated in mice fed with 0.5% 3′SL diet (Figure 2A). This was explained by a rise in acetate (Figure 2B), propionate (Figure 2C) and butyrate (Figure 2D), which was not observed for the other dietary intervention groups. Levels of iso-butyrate, valerate and iso-valerate in cecum content were below detection limit and are therefore not individually displayed.

### 3.3 Dietary 3′SL limits HDM-induced pulmonary influx of immune cells

**Figure 3:**
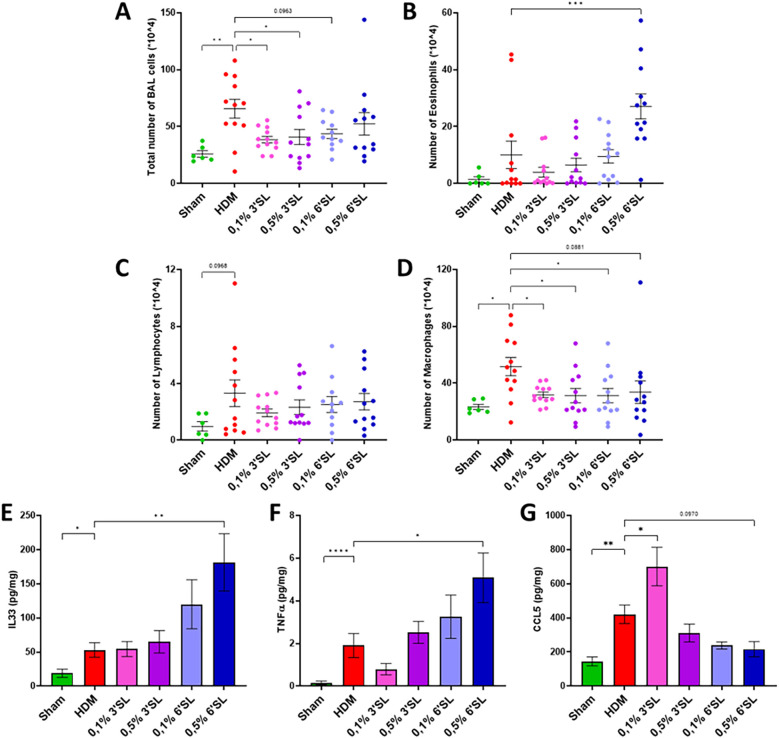
Influx of inflammatory cells in the BALF of mice. **(A)** The total cell count in BAL fluid, **(B)** eosinophils, **(C)** lymphocytes and **(D)** macrophages are displayed. Furthermore, presence of proinflammatory cytokines and chemokines **(E)** IL33, **(F)** TNFα and **(G)** CCL5 in lung tissue homogenate supernatants were quantified. Data is presented as mean ± SEM of *n* = 6 (sham) or *n* = 12 (allergic) animals per group. Sham and HDM-allergic groups were statistically compared using an unpaired *t*-test. Dietary intervention groups were compared to the HDM-allergic group by One-Way ANOVA followed by a Dunnett's *post hoc* test (*p < 0.05, **p < 0.01, ***p < 0.001).

BALF cell counts were determined to assess influx of inflammatory cells into the airways 72 h after the final HDM challenge. The total number of BALF cells was increased in the HDM sensitized mice compared to Sham (Figure 3A). Both 3′SL supplemented diets largely prevented a rise in BALF cell influx in HDM sensitized and challenged mice compared to mice receiving the control diet. The HDM sensitized and challenged mice did not show a significant increase in eosinophil influx compared to Sham mice (Figure 3B), but HDM sensitized mice fed the 0.5% 6′SL had a significantly higher number of eosinophils present in BALF compared to HDM sensitized and challenged mice on control diet. The number of lymphocytes was not significantly changed in any of the groups (Figure 3C). Yet, HDM sensitized and challenged mice fed control diet had an increased influx of macrophages into the airways compared to Sham mice. All dietary intervention groups showed a reduced macrophage influx in the BALF (Figure 3D). Influx of immune cells in inflamed tissue is a consequence of chemoattraction upon increased secretion of proinflammatory cytokines and chemokines, such as IL33, TNFα and CCL5. Levels of IL33 and TNFα (Figures 3E,F) in lung homogenate supernatants were found to be further increased in HDM sensitized and challenged mice receiving a 0.5% 6′SL supplemented diet, while CCL5 levels tended to be decreased (*p* = 0.0970, Figure 3G). These data show that dietary 3′SL, but not 6′SL, lowers macrophage influx, while mice fed 0.5% 6′SL have higher levels of proinflammatory cytokines present in lung tissue.

### 3.4 Dietary 6′SL decreases the percentage of regulatory T cell subsets in lung tissue

**Figure 4:**
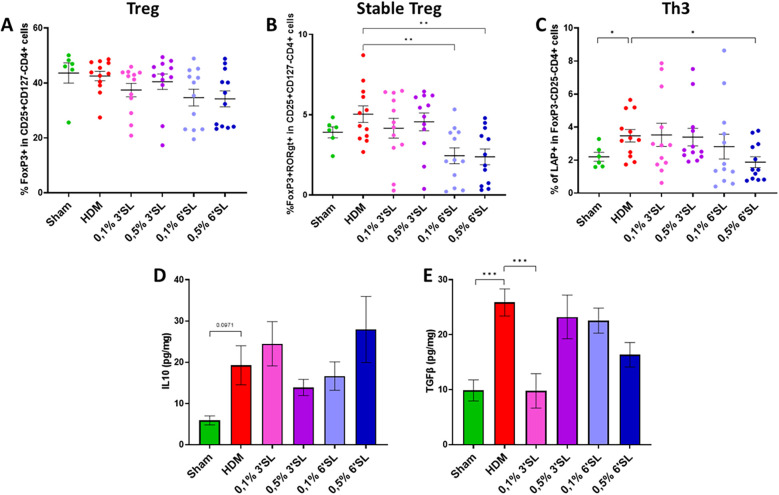
Populations of **(A)** Treg, **(B)** stable Treg and **(C)** Th3 cells were determined by flow cytometric analysis of single cell suspensions obtained from collected lung tissue. Normalized levels of **(D)** IL10 and **(E)** TGFβ were determined in lung homogenate supernatants. Data is presented as mean ± SEM of *n* = 6 (sham) or *n* = 12 (allergic) animals per group. Sham and HDM-allergic groups were statistically compared using an unpaired *t*-test. Dietary intervention groups were compared to the HDM-allergic group by One-Way ANOVA followed by a Dunnett's *post hoc* test (*p < 0.05, **p < 0.01, ***p < 0.001).

To further assess the immunomodulatory effects of dietary 3′SL and 6′SL on HDM-induced allergic asthma development, the ratio of regulatory T cell subsets and regulatory cytokines in lung tissue was assessed. The percentage of regulatory T (Treg, FoxP3 + in CD25 + CD127 − CD4+) cells was not altered in any of the groups (Figure 4A). Although HDM sensitized and challenged mice had a similar percentage of stable Treg (FoxP3 + RORγt + in CD25 + CD127 − CD4+) cells compared to Sham mice (Figure 4B), HDM sensitized and challenged mice receiving a 6′SL supplemented diet had a significantly lower proportion of stable Tregs present in lung tissue compared to HDM sensitized and challenged mice receiving control diet. In addition, the population of regulatory type Th3 (LAP + in FoxP3 − CD25 − CD4+) cells was determined and was found to be more abundant in HDM sensitized and challenged mice compared to Sham mice fed the control diet and remained increased in HDM mice fed the 0.1% or 0.5% 3′SL diet or 0.1% 6′SL diet (Figure 4C). The presence of these regulatory Th3 cells was, however lower in HDM sensitized and challenged mice receiving the 0.5% 6′SL diet when compared to the control diet. Effector functions of these regulatory cells depend in part on the secretion of the regulatory cytokines IL10 and TGFβ (Figures 4D,E). In the control diet fed HDM allergic mice the pulmonary IL-10 levels tended to increase compared to sham controls, this was not significantly affected by the HMOS diets. TGFβ was also increased in HDM sensitized and challenged mice fed control diet, while TGFβ was normalized in HDM mice receiving 0.1% 3′SL diet. These data indicate that dietary 6′SL, but not 3′SL, lowers the abundance of several pulmonary Treg cell subsets.

### 3.5 Dietary 3′SL and 6′SL affect pulmonary levels of IL13 and IFNγ

**Figure 5:**
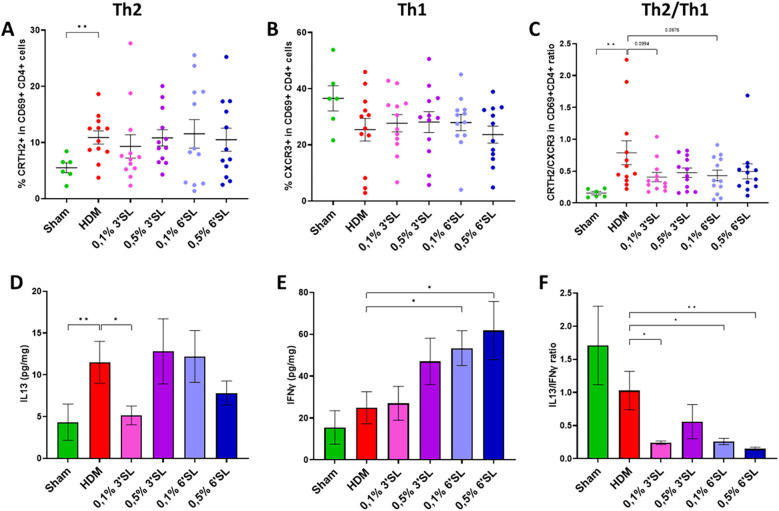
Single cell suspensions were obtained from collected lung tissue for flow cytometric analysis. Populations of **(A)** activated Th2 cells, **(B)** activated Th1 cells and **(C)** the ratio of Th2/Th1 cells were determined. Levels of **(D)** IL13, **(E)** IFNγ and **(F)** IL13/IFNγ ratio were determined in lung homogenates. Concentrations were normalized per mg of lung tissue. Data is presented as mean ± SEM of *n* = 6 (sham) or *n* = 12 (allergic) animals per group. Sham and HDM-allergic groups were statistically compared using an unpaired *t*-test. Dietary intervention groups were compared to the HDM-allergic group by One-Way ANOVA followed by a Dunnett's *post hoc* test (*p < 0.05, **p < 0.01).

To further phenotype the allergic type 2T cell response in the lung tissue, the presence of Th1 and Th2 type cytokines and percentage of activated T helper cell subsets was examined. Total Th1 and Th2 populations are shown in Supplementary Figures S3B,C. An increased percentage of activated Th2 (T1ST2 + in CD69 + CD4+) cells and elevated levels of IL13 were observed in HDM sensitized and challenged mice (Figures 5A,D). HDM sensitized and challenged mice fed a 0.1% 3′SL had reduced levels of IL13 in long homogenate supernatants compared to HDM sensitized and challenged mice fed control diet. However, a decrease in the percentage of activated Th2 cells was not observed. Furthermore, presence of activated Th1 (CXCR3+ in CD69 + CD4+) cells was not altered upon HDM sensitization and challenge, shifting the Th2/Th1 cell balance in favour of the Th2 cells in HDM sensitized and challenged mice receiving control diet (Figures 5B,C). None of the groups receiving an HMOS diet had an altered percentage of activated Th1 cells or change in the Th2/Th1 cell ratio. However, HDM sensitized and challenged mice receiving 6′SL diets showed a significant increase in IFNγ concentration in lung homogenate supernatants (Figure 5E). The ratio of IL13/IFNγ present in lung tissue was significantly lower in groups receiving 0.1% 3′SL, 0.1% 6′SL or 0.5% 6′SL diets compared to HDM sensitized and challenged mice fed control diet (Figure 5F). Therefore, these data show immunomodulation by dietary 3′SL and 6′SL on the levels of cytokine secretion in the lungs steering away from the type 2 signature.

### 3.6 Dietary 3′SL reduces HDM-IgE serum levels and mucosal mast cell degranulation

**Figure 6:**
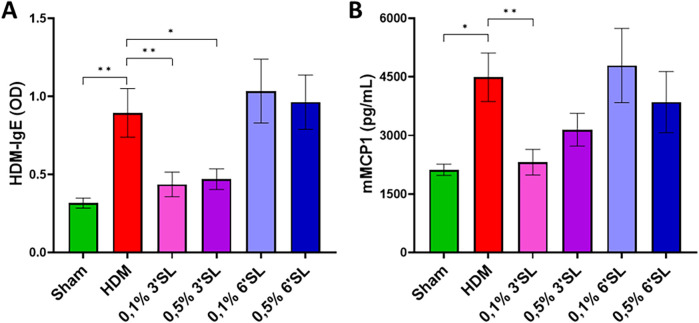
**(A)** Serum levels of HDM-specific IgE and **(B)** mMCP1 were measured. Data is presented as mean ± SEM of *n* = 6 (sham) or *n* = 12 (allergic) animals per group. Sham and HDM-allergic groups were statistically compared using an unpaired *t*-test. Dietary intervention groups were compared to the HDM-allergic group by One-Way ANOVA followed by a Dunnett's *post hoc* test (*p < 0.05, **p < 0.01).

During allergen sensitization, allergen-specific IgE is produced which can be measured in serum. HDM sensitized and challenged mice receiving control diet had higher levels of HDM-specific IgE (Figure 6A) as well as HDM-specific IgG1 and IgG2a (Supplementary Figures 3I,J) present in serum. Dietary intervention with 3′SL largely prevented this increase in HDM-IgE levels. This was not observed in mice receiving a 6′SL containing diet. During allergen challenge, mucosal mast cells degranulate upon IgE crosslinking and proinflammatory mediators are released. One of these mediators, murine mast cell protease 1 (mMCP1), was measured in serum to reflect mucosal mast cell degranulation (Figure 6B). mMCP1 was elevated in HDM sensitized and challenged mice compared to Sham mice. HDM sensitized and challenged mice fed a 0.1% 3′SL diet had a significantly lower level of mMCP1 in serum compared to HDM sensitized and challenged mice on control diet; this was not observed for the other dietary intervention groups. These data indicate that 3′SL, but not 6′SL, is capable of preventing HDM sensitization in this murine model for acute HDM induced allergic asthma.

## 4 Discussion

Sialylated HMOS are found in abundance in human milk and are therefore thought to fulfill biological functions during neonatal development (17, 18). These sialylated HMOS are described to possess immunomodulatory effects (30, 36) and modulate immune function in allergic settings (34, 37). These effects may be partially explained by the presence of sialic acid groups. Previously dietary supplementation with several prebiotic structures like short chain (sc) GOS and long chain (lc) FOS, which do not contain sialic acid groups, and their metabolites improved asthma allergic outcomes in mice (28, 32, 38–40). A recent study showed protective effects of both 2FL and 6′SL (500 mg/kg/day via oral gavage or drinking water) in prevention of HDM allergic asthma in mice when started directly after weaning (33). The aim of this study was to compare the efficacy of 3′SL and 6′SL via dietary supplementation on the development of HDM-induced acute allergic asthma in early life (6 weeks old mice). Airway hyperresponsiveness and immunological parameters were determined to assess the severity of the allergic airway inflammation.

In this study, HDM sensitized and challenged mice receiving the 0.5% 3′SL, 0.1% 6′SL or 0.5% 6′SL containing diets exhibited an attenuated increase in airway hyperresponsiveness which is a general disease feature in allergic asthma patients. Previously, it was shown that prebiotic fibers improved HDM-allergic outcomes in a similar preclinical HDM-induced acute allergic asthma model (32). The methacholine induced AHR response in the current study, however was relatively mild and only gained significance when calculating the AUC of AHR in HDM allergic mice compared to controls. Future studies should therefore confirm the protective effect of 3′SL and 6′SL in a more severe AHR HDM model. Prebiotic fibers are fermented into SCFAs by intestinal microbes. SCFAs analysis provides a functional readout of microbial function, serving as a proxy for changes in microbial community composition. SCFAs are potent immunomodulators, known to affect e.g., different types of leukocytes promoting homeostasis (41). Administration of the SCFA propionate was previously found to improve HDM allergic asthma outcomes, including airway hyperresponsiveness, in mice (28). In addition to propionate, increased butyrate concentrations may also contribute to ameliorating allergic inflammation and clinical symptoms (42). In the current study, mice receiving the 0.5% 3′SL supplemented diets contained higher concentrations of SCFAs, including propionate in the cecum content, and demonstrated decreased airway hyperresponsiveness. On the other side, mice receiving 6′SL supplemented diets also demonstrated a reduction in airway hyperresponsiveness, while the SCFAs levels in these mice were not elevated significantly. Additionally, the 0.1% 6′SL fed mice contained increased cecal propionate levels compared to control diet fed HDM allergic mice (*p* = 0.0731). This indicates that bacterial metabolites, such as propionate, may have contributed to the protection against airway hyperresponsiveness in HMOS fed HDM allergic mice (43). Since we have observed differences in cecal SCFAs concentration between HMOS fed groups, future studies should explore how sialylated HMOS affect microbiome composition. In addition, future studies could focus on studying the underlying mechanism by which 3′SL and 6′SL lower methacholine induced AHR, by means of histological analysis of lung tissue.

A lower influx of leukocytes, especially macrophages, into the lungs was observed in mice receiving the 3′SL containing diet. Prebiotic dietary interventions using scGOS led to a reduction in total influx of cells into the lungs (32, 39), which was observed in this study as well. Mice fed scGOS containing diets had lower numbers of eosinophils present in broncho alveolar lavage (32, 39). However, in the current study, the airway eosinophilia was not significantly increased in the HDM allergic mice but all other relevant type 2 markers on allergic airway sensitization were present. The decrease in total cell influx could mainly be explained by lower numbers of macrophages, which was observed in both the 3′SL intervention groups, but also in the 0.1% 6SL group. The rise in BALF number of monocytes/macrophages during allergic inflammation may be a reflection of proliferation of local alveolar and interstitial macrophages and/or an increase in newly attracted bone marrow derived monocytes which further differentiate into macrophages in the lung tissue (44). 3′SL or 6′SL may suppress local tissue macrophage proliferation and M2 phenotype development for example by affecting the HIF1alpha/VEGF axis or via blocking TLR4 linked epigenetic activation (45, 46). Furthermore, inhibition of the β-catenin/CBP pathway may have resulted in selective lowering of macrophage type inflammation (47). Future studies should focus on mechanisms underlying the reduced monocyte/macrophage inflammation in the 3′SL or 6′SL fed HDM sensitized and challenged mice.

Even though mice receiving the 0.5% 6′SL diet showed improved clinical outcomes, an increase in eosinophil influx into the lungs was observed. Secretion of IL33 by airway epithelial cells is a key phenomenon in allergic asthma (48). Neutralization of IL33 was found to reduce the influx of eosinophils into the lungs of mice (49), revealing a link between IL33 and eosinophilic inflammation in the lungs dependent on systemic IL5 secretion (50). Here, the increased eosinophilic influx in the lungs was associated with elevated IL33 levels. The presence of IL33 promotes the release of TNFα from macrophages and local mast cells (48), and TNFα in turn promotes airway epithelial cells to secrete IL33 (51). Dietary 0.5% 6′SL therefore may have induced IL33 and/or TNFα secretion by local tissue inflammatory cells. It should be noted that these effects are dose related, since 0.1% 6′SL did not show increased pulmonary IL33 or TNFα levels, nor increased airway eosinophilia. Although the general idea is that both eosinophils and IL33 are related to increased inflammation, studies have demonstrated that these markers are also involved in regulatory responses and may therefore contribute to alleviating clinical symptoms (52, 53). Additionally, sialylated HMOS show affinity for sialic acid-binding immunoglobulin-like lectins (Siglecs), these receptors are expressed on various immune cells such as dendritic cells, macrophages and eosinophils, and are known to mediate dampening of inflammatory responses (16). Hence apart from microbiome modulation, HMOS are known to become available in the blood stream (54) and may also directly have affected immune cell properties. Especially 6′SL may interact with several Siglecs (55), which may have contributed to lowering clinical symptoms.

Sialylated HMOS are known to possess binding affinity to the TLR4 receptor (31). HDM allergens are known to activate bronchial epithelium via direct binding to TLR4 or proteolytic activity indirectly activating TLR4 (56, 57). Furthermore, HDM allergens can cross the epithelial barrier via disruption of tight junctions (58) and become systemically available for immune cells. Activation of T cells via TLR4 induces TNFα secretion, which is reduced by Th3 cells (59). In neonatal mice, 6′SL supplemented formula milk reduced TNFα gene expression partially via direct binding to and inhibition of TLR4 (60). Yet, in a murine model for colitis, 3′SL supplementation promoted dendritic cell function in a TLR4-dependent manner (31). 3′SL or 6′SL may become available systemically, which is known for specific HMOS structures, and occupy the TLR4 receptor in this way hampering the HDM-induced activation cascade. While both 3′SL and 6′SL indeed are known to modify mucosal immunity via TLR4 interaction, this present study clearly demonstrated differences in immunological outcomes in mice fed a 3′SL or 6′SL containing diet in this HDM-induced acute allergic asthma model. The mice receiving dietary 3′SL had lower airway inflammation, while dietary 0.5% 6′SL enhanced eosinophilic airway inflammation. Differential TLR4 binding and signaling features of 3′SL vs. 6′SL may play a role in the dose dependent differences of effect observed between these HMOS.

CCL5 (also known as RANTES) is a chemokine secreted by bronchial epithelial cells (61) and fibroblast from asthmatic individuals (62) and is known to attract several immune cells, including T cells and eosinophils (61). Yet, this chemokine can drive a Th2 response towards a Th1 response (63) and the presence of CCL5 is linked to a decreased airway hyperresponsiveness during repeated allergen exposure (64). However CCL5 has been linked to the development of fibrosis as well (65). Furthermore, TGFβ is considered a regulatory cytokine, known to generally dampen immune responses. On the contrary, this growth factor is also involved in fibrosis during asthma (66). High levels of TGFβ in the lungs are therefore associated with markers of increased airway remodeling (67, 68). 3′SL or 6′SL differentially affect the balance between TGFβ and CCL5. In particular in the 0.1% 3′SL fed HDM mice, CCL5 significantly increased, while TGFβ reduced. Only the 0.1% 3′SL diet could not protect against development of airway hyperreactivity, while 0.5% 3′SL or 0.1% and 0.5% 6′SL could. Future studies are warranted to understand the relevance of the balance between TGFβ and CCL5 in the development of HDM induced loss in airway function.

In addition to the aforementioned effects, 3′SL and 6′SL may have affected the development of Treg cells. The population of both TGFβ producing pulmonary stable Treg and Th3 cells were lower only in mice fed the 0.5% 6′SL diet, but not affected in the other dietary interventions groups. Therefore, the populations of Treg cells do not provide an explanation for the improved clinical outcomes. In addition, the clinical improvement cannot be explained by the percentage of Th2 and Th1 cells present in lung tissue either, even though the balance of Th2/Th1 in the mice fed the 0.1% 3′SL or 0.1% 6′SL diet tended to shift in favor of the Th1 cells. These effects became more pronounced when focusing on Th2 and Th1 cytokines present in the lung tissue. In all HMOS diet groups the balance was shifted towards type 1 (based on IFNγ) and away from type 2 (based on IL13) when compared to HDM sensitized and challenged mice receiving the control diet. Mice receiving the 0.1% 3′SL diets had significantly lower levels of IL13 in lung tissue. Yet, the mice fed a 0.1% or 0.5% 6′SL supplemented diet were found to have increased levels of IFNγ present in lung tissue, shifting the balance towards Th1 dominant cytokine production, which may have counteracted the effect of type 2 cytokines in chronic inflammation and airway hyperresponsiveness (69).

Furthermore, mice receiving 0.1% or 0.5% 3′SL, but not 6′SL containing diets had lower levels of HDM-specific IgE and mMCP1 in serum. Simultaneously, an increase in HDM-IgG1 and HDM-IgG2a was observed in HDM-sensitized mice, which were not altered in mice receiving an HMOS supplemented diet. Upon HDM exposure, mucosal mast cells opsonized with HDM-IgE can be active and release mMCP-1 which can be measured in the serum. Since 3′SL prevented HDM-IgE sensitization, this may have resulted in reduced mast cell activation upon HDM challenge. Activation of mast cells can also be prevented by SCFAs (70), however only in mice fed the 0.5% 3′SL diet SCFA levels were enhanced in the cecum and local tissue levels may not be sufficient to reduce mast cell activation. Therefore, it can be hypothesized that 3′SL may have acted upstream in the sensitization cascade, suppressing the HDM-induced activation of bronchial epithelial cells and/or dendritic cells and macrophages, leading to lesser type 2 immunity and HDM sensitization (71). These findings indicate that future studies should elaborate on the exact mechanisms and pathways involved in interaction between the immunomodulatory effects of sialylated HMOS and the development of allergic asthma. To the best of our knowledge, this is the first study providing a direct comparison between these abundantly present sialylated HMOS both dosed 0.1 and 0.5%. This observational and explorative murine study provides several leads for future mechanistic pathways which sialylated HMOS exert immunomodulatory effects. The efficacy of 3′SL and 6′SL should be further investigated in chronic models of HDM-induced allergic asthma. Furthermore, this study reinforces the immunological benefits of HMOS present in breastfeeding in shaping immune function. Although the precise mechanisms and magnitude of its allergy-preventive effects remain to be further elucidated, these findings provide a basis for further investigation into targeted nutritional interventions in mitigating (allergic) inflammation.

## 5 Conclusion

In conclusion, this study demonstrated that dietary 3′SL and 6′SL differentially affect clinical and immunological outcomes in HDM sensitized and challenged mice. Mice receiving the 0.5% 3′SL diet showed increased cecal SCFA levels and lowered HDM sensitization, airway hyperresponsiveness and allergic airway inflammation. Mice receiving 6′SL containing diets also exhibited a smaller increase in airway resistance, but the airway inflammation was enhanced in the 0.5% 6′SL fed mice. Both 3′SL and 6′SL were found to lower BALF macrophage counts. Future studies should focus on elucidating the underlying mechanism via which sialylated HMOS exert their preventive effect in the airway allergic response.

## Data Availability

The raw data supporting the conclusions of this article will be made available by the authors, without undue reservation.
