# Supplementary material for: Dietary 3'-sialyllactose reduces sensitization and type 2 inflammation in a house dust mite induced acute allergic asthma model
Source: Front Allergy. 2025 Dec 1;6:1604917. doi: 10.3389/falgy.2025.1604917 (PMC12702718; doi:10.3389/falgy.2025.1604917)
Supplement: Supplementary file 1 [file Supplementaryfile1.docx]

Supplementary Material

# Supplementary Figures and Tables

## Supplementary Figures


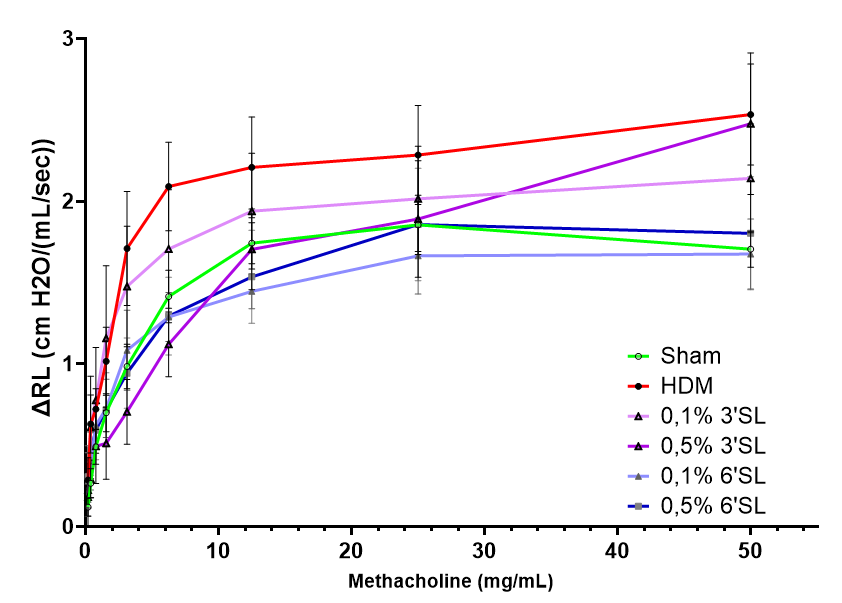


Supplemental figure 1. The increase in airway resistance in response to progressive doses of methacholine was measured in ventilated anesthesized mice. Metacholine concentrations (0, 0.39, 0.78, 1.56, 3.13, 6.25, 12.5, 25 and 50mg/mL) are displayed on a linear axis.


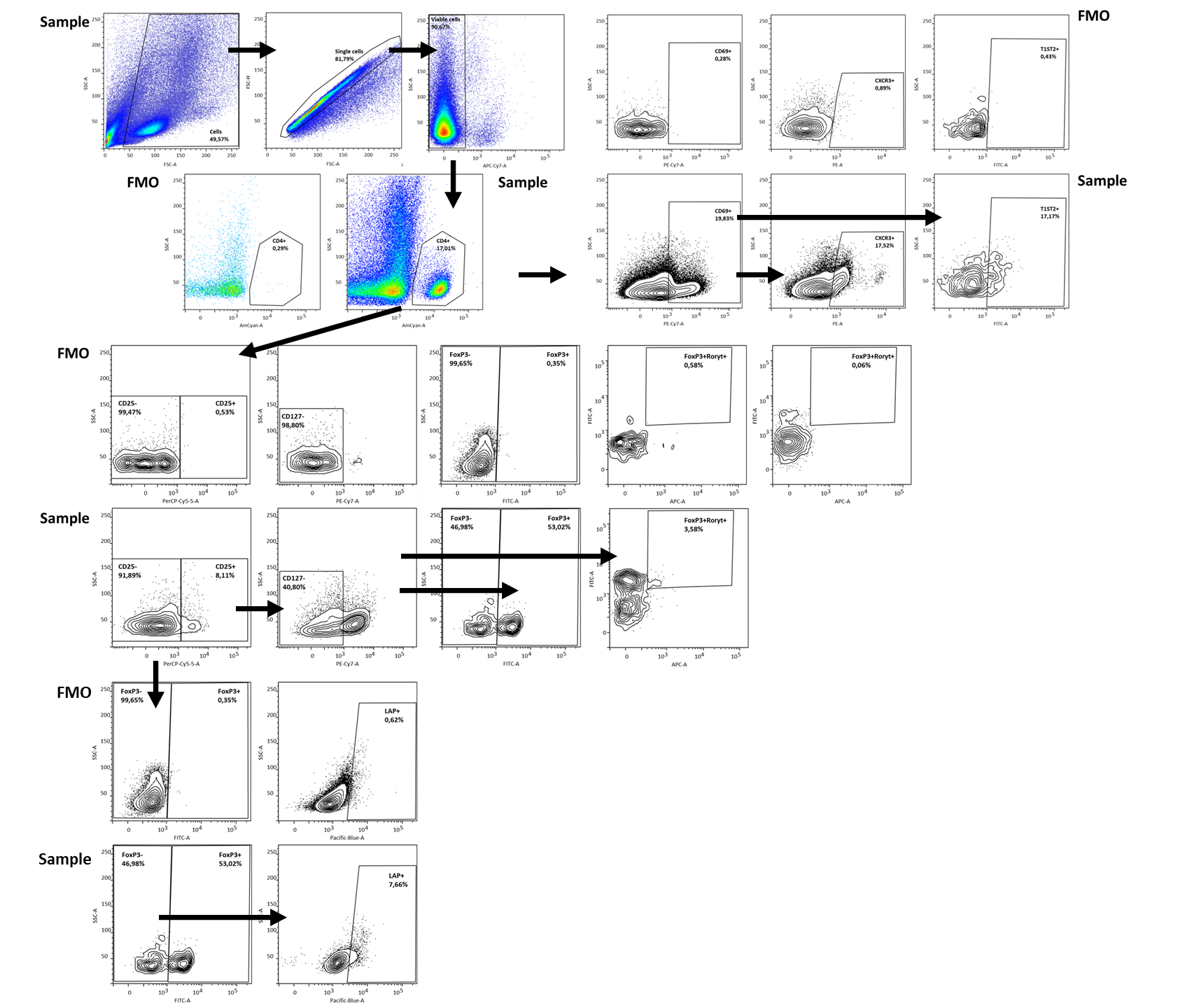


Supplemental figure 2. Representative gating strategy, including corresponding FMOs for flow cytometric analysis of single cells suspensions obtained from lung tissue.

Supplemental figure 3. A) Influx of neutrophils was quantified in BALF. Percentages of T helper subsets present in lung tissue were determined by flow cytometry. Populations of B) Th2 (T1ST2+ in CD4+) cells, C) Th1 (CXCR3+ in CD4+) cells and D) Th17 (CCR6+RORγt+ in CD4+) cells were determined. Furthermore, levels of E) CCL20, F) CCL22, G) IL5 and H) IL17 per mg lung tissue were measured by ELISA. Serum I) HDM-specific IgG1 and J) HDM-specific IgG2a levels were measured by ELISA. Data is presented as mean ± SEM of n=6 (sham) or n=12 (allergic) animals per group. Sham and HDM-allergic groups were statistically compared using an unpaired t-test. Dietary intervention groups were compared to the HDM-allergic group by One-Way ANOVA followed by a Dunnett’s post hoc test (* p< 0.05, ** p< 0.01, *** p,< 0.001, **** p< 0.0001).
